# A Robust RANSAC-Based Planet Radius Estimation for Onboard Visual Based Navigation

**DOI:** 10.3390/s20144041

**Published:** 2020-07-21

**Authors:** Francesco de Gioia, Gabriele Meoni, Gianluca Giuffrida, Massimiliano Donati, Luca Fanucci

**Affiliations:** 1 Department of Information Engineering, University of Pisa Via Girolamo Caruso 16, 56122 Pisa, PI, Italy; gianluca.giuffrida@phd.unipi.it (G.G); massimiliano.donati@unipi.it (M.D.); luca.fanucci@unipi.it (L.F.); 2 IngeniArs S.r.l; Via Ponte a Piglieri 8, 56121 Pisa, PI, Italy; gabriele.meoni@ingeniars.com

**Keywords:** RANSAC, visual based navigation, optical navigation, radius estimation, circle fitting, image processing

## Abstract

Individual spacecraft manual navigation by human operators from ground station is expected to be an emerging problem as the number of spacecraft for space exploration increases. Hence, as an attempt to reduce the burden to control multiple spacecraft, future missions will employ smart spacecraft able to navigate and operate autonomously. Recently, image-based optical navigation systems have proved to be promising solutions for inexpensive autonomous navigation. In this paper, we propose a robust image processing pipeline for estimating the center and radius of planets and moons in an image taken by an on-board camera. Our custom image pre-processing pipeline is tailored for resource-constrained applications, as it features a computationally simple processing flow with a limited memory footprint. The core of the proposed pipeline is a best-fitting model based on the RANSAC algorithm that is able to handle images corrupted with Gaussian noise, image distortions, and frame drops. We report processing time, pixel-level error of estimated body center and radius and the effect of noise on estimated body parameters for a dataset of synthetic images.

## 1. Introduction

Future deep-space exploration is expected to rely more and more on autonomous spacecraft [1,2,3,4]. Indeed, traditional ground-based radio navigation poses limitations in terms of data transmission latency, channel availability, and robustness to communication failures. Thus, new emerging technologies support the transition to autonomous spacecraft. Among these, optical navigation is considered to be one of the most promising solutions, as Charge-coupled device (CCD) and Complementary Metal Oxide Semiconductor (CMOS) cameras are usually available on modern spacecraft [4,5,6,7,8,9,10]. The main objective of optical navigation systems is to process raw images acquired by the camera, in order to obtain measures and information for navigation. Typical information required for visual-based navigation is the Line-Of-Sight (LOS) vector obtainable as a function of the celestial body centroid and radius. These measurements are further processed by navigation filters that include the dynamic of the system to perform autonomous trajectory adjustments. This class of image processing algorithms may require significant computational resources that may not be available onboard. As reported in [11], missions such as NEAR Shoemaker and Hayabusa spacecraft overcome this limitation by sending images to be processed to ground. Clearly, this solution has the same drawbacks as traditional radio tracking, thus new optical navigation methods are designed to account for the specific onboard computational capabilities. In addition, different navigation phases (approaching a distant celestial body, collaborative/non-collaborative rendezvous, and landing) require different measurements at different levels of precision. Implementation of a general navigation system that is able to operate in all of these scenarios is a challenging problem and requires a consistent amount of computational resources that might be unavailable onboard.

In this paper, we address the problem of estimating the centroid and radius of a celestial body from a single raw grayscale image taken by an onboard camera. These estimates can be used to derive the relative distance between the celestial body and the approaching spacecraft. Different sources of noise in an Autonomous Visual Based Navigation pose serious problems as they can potentially introduce large errors in the navigation system. Various works present methods that are designed to cope with numerous families of noise sources. In this work, we provide a novel solution based on a pre-processing pipeline and a final model estimation designed to be robust to three very dissimilar sources of noise—Gaussian noise, image distortion, and frame drop. We developed a multi-step procedure based on Random Sample Consensus (RANSAC) [12] designed to be implemented in small onboard computers. Our approach is able to cope with various amounts of noise and frame alterations (scanline drop or distortions). We measured the effect of such sources of noise on the final estimation for a dataset of synthetic images. The rest of this paper is organized as follows. In Section 2, we summarize the state-of-the-art methods and approaches in solving autonomous visual navigation problems. In Section 3 we detail our image processing pipeline. In Section 4 we report the estimation accuracy of our system and analyze robustness to various sources of noise. In Section 5 we discuss the possible scenario of application and possible improvements for future works.

## 2. Previous Works

The problem of Autonomous Navigation Systems for spacecraft navigation has been long studied and various approaches have been proposed [3,13]. An emerging set of solutions to this application are based on Image processing techniques to estimate the relative position between the starcraft and the target body. Such techniques can be implemented for onboard computers in modern spacecraft since they require only an image acquisition system. Currently, there is no systematic way to validate these systems, although authors usually test their methods on a simulated dataset or real images acquired by previous missions [11].

The problem of estimating the radius of a celestial body is usually divided into three phases. First, the target is isolated from the deep-space background (segmentation), then the points that lie on the target limb are filtered from the points that are inside the target and those that lie near the terminator (the line separating the illuminated face from the backlit face) [14]. The third phase is a model fitting algorithm on the selected set of points. The output is the set of parameters describing the best fitting model for the set of points. For celestial bodies such as planets and spacecraft, models are generally ellipses; however, if the oblateness (the eccentricity) of the target is negligible, ellipses can be replaced by circles that are computationally simpler to handle. In this paper, we assume a circle model fitting algorithm where the circle is fully described by their center coordinates and their radius.

In [7] image differentiation is obtained with a fourth order Richardson extrapolation, then outliers are removed with box-based method and RANSAC. The elliptical model parameters are obtained with Taubin curve fitting [15] algorithm and Circular Sigmoidal Function (CSF) Least Squares model estimation. The Circular Sigmoidal Function fitting is also used in [8] where a camera-only attitude estimation algorithm is described. The algorithm approximates the radius ellipses using a computationally inexpensive Least Squares fit of sigmoidal functions that achieves high accuracy even for smooth limb transition. In [11], a pre-processing phase performs threshold segmentation, background noise filtering, and image sharpening before Canny edge detection. Pseudo-edges (points lying on the terminator) are removed selecting the edges aligned with the direction of the light source. A curve model fitting is then applied to the remaining points. A comparison of Least Squares and Levenberg–Marquardt ellipse-fitting is reported. This approach performs well on targets with different texture and in different lighting conditions; however, it relies on the knowledge of the lighting direction. In [6], the authors developed a full-disk image processing pipeline to estimate the camera-to-moon relative position assuming a pinhole camera model. The target is segmented from background using thresholding and limb pixels are obtained using first a Canny edge detector. Background noise is removed through a noise removing filter after threshold segmentation. The method requires the direction of the light source that can be retrieved from an external sensor or inferred from further processing. As reported, the procedure has an unpredictable error in case of scarce target illumination. In [9], the authors describe a camera-based three-axis attitude sensor based on the estimation of the spacecraft-to-Moon direction. An image processing pipeline processes a single grayscale image and estimates the Moon center coordinates with a Least Squares estimator minimizing the standard deviation instead of the mean squared. This approach is reported to be less sensitive to spurious points that do not lie on the body edge. In [10], the target is segmented performing thresholding, then a morphological opening operation is applied to remove noise, secondary objects, and background stars. A rectangular Region Of Interest (ROI) is described around the target to reduce the computational demand of the next steps. Edges are extracted with a Prewitt filter and pseudo-edges are removed using Zernike moments. Final parameter estimation for elliptical model fitting is performed using the M-Estimator Sample Consensus (MSAC) algorithm.

In [16], the authors propose a multi-step image processing pipeline for optical navigation. In their pipeline authors include non-local filtering, morphological opening, and gradient direction processing to pre-process the input image and obtain relevant edges. Final centroid and radius estimation is performed with Least Squares. In the paper, the authors do not take into account other sources of noise other than Gaussian noise. In our work, we extend the analysis including image distortion and frame drop. In [17], authors derive a mathematical model for a nanosatellite and implement an Extended Kalman Filter to support Autonomous Optical Navigation. In [18], authors describe a flexible and easy to implement interplanetary autonomous optical navigation system. The algorithm is able to use images of two celestial bodies to improve positioning and achieving high accuracy in noise-free images.

In [6,9,11], the authors used the Canny edge detector algorithm as a mean to obtain a cleaner thin edge. We preferred to reduce the computational complexity by using the Prewitt edge filter and by moving the outlier filtering in the last steps of the processing pipeline. The RANSAC algorithm has been already used in [8,14] to estimate celestial bodies radius. In our work, we introduced an edge mask based on morphological operations and an online model validation by sector binning. We also tested the robustness of our approach to various sources of noise (Gaussian noise, scanline drop, and frame alteration).

## 3. Methods

We propose a multi-step method to estimate the radius of a circular-shaped celestial body from a single channel grayscale image. In this section, we will refer to the celestial body as the *target*.

**Figure 1:**
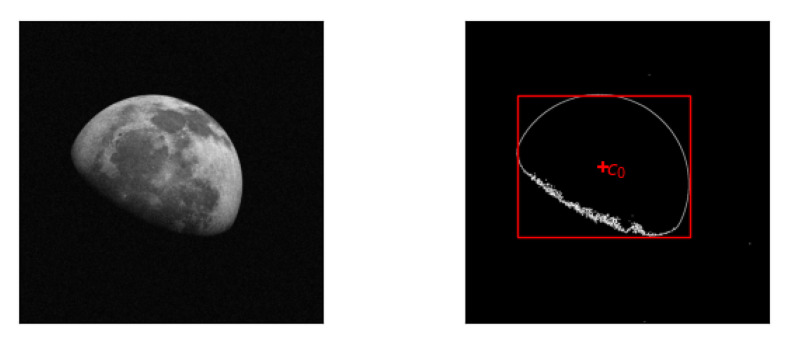
Output of the first step of the pipeline. The detected bounding box is a first crude estimation of the target centroid $c_{0}$.

In the first step, we threshold the image with a tunable threshold value $\tau_{1}$, as we expect the background to be much darker than the target. In the thresholded binary image, the target appears as a blob. We can find the blob bounding box and obtain a first crude approximation of the target center $c_{0}$ using the marching squares algorithm [19,20]. A graphical representation of the output for this first step is given in Figure 1. Optionally, we can also use the bounding box to define a rectangular Region Of Interest (ROI) around the target, thus reducing the original image size, if needed.

In the second step, we apply an edge detection filter to identify the sharp transition from the dark background to the illuminated target. We approximate the image gradient with a discrete filter, such as Sobel [21] or Prewitt [22]—we observed small differences in applying these two filters, thus we consider them to be equivalent. In our method, we considered only the image gradient magnitude, as we are not interested in the direction of the image gradient.

The third step applies dilation (⊕) and erosion (⊖) operations to the binary image in order to produce a thick ring-shaped mask around the target borders. The edge mask is computed as:

(1) edge_mask=M∧¬m

where ∧ and ¬ are the logical AND and NOT operations, respectively, $m=\left(I>\tau_{1}\right)⊖1_{3x3}$, $M=m⊕1_{5x5}$ with $1_{3x3}$ and $1_{5x5}$ as square structuring elements.

We use this edge mask to select only the pixels from the edge image that lie at blob boundaries. This approach removes regions with high frequency components located inside the target boundary, such as regions with irregular texture (craters) or inconsistent lighting conditions. The magnitude values of the masked edges are further compared with a threshold value $\tau_{2}$ in order to remove edges resulting from high contrast near the terminator. Indeed, we observed that stronger edges belong to the real target border, whereas edges with smaller values belong to the shaded area. We notice that simple thresholding is sufficient to eliminate most of the edges belonging to the shaded area; the remaining edges do not interfere much in the target radius estimation, as they are expected to be marked as outliers by RANSAC and suppressed accordingly. At the end of step 3, we produce as output the list of (x,y) image coordinates corresponding to the remaining edges after thresholding.

**Figure 2:**
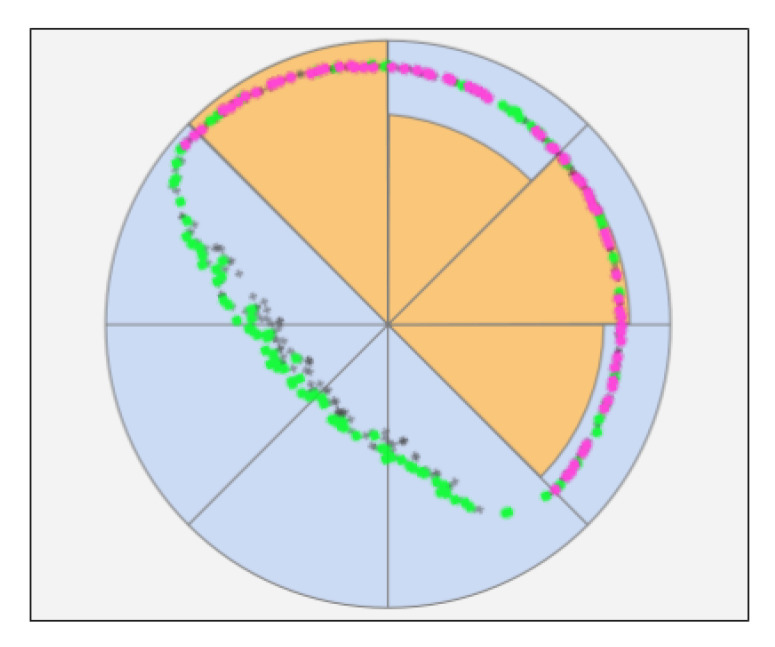
Graphical representation of sector binning. In this example, the *K* parameter is set to 8 and 4 sectors are active. The orange circular sectors represent the number of points that lies inside the corresponding sector.

In the fourth step, we transform the (x,y) image Cartesian coordinates to (ρ,θ) polar coordinates by using the target center $c_{0}$ as the origin. Next, we divide the angles in *K* bins and define an *active bin* as a bin with more than *N* points in it. We discard images that have less than *S* out of *K* active bins and preserve images that have at least *S* out of *K* active bins Figure 2.

We used this technique to filter images with points scattered along a circumference, from images that contain points clustered tightly along a short arc, since the first images produce better radius estimations than the latter. For each bin, we select the first points with the largest radius values in order to cope with images that present thin limbs (we refer to it as “sickle effect”). In this kind of image, the gradient has high values both on the true target border and on the terminator, thus points form a sickle-shaped curve that RANSAC can arbitrarily fit with a model for the outer circle or a model for the inner circle. By selecting points with a higher ρ value, we force RANSAC to estimate the model for the outer circle.

In the fifth and last step, we apply a RANSAC procedure for radius estimation. The algorithm returns the best fitting circle model (as center and radius) and the set of inliers points. Compared to a Least Squares estimator, RANSAC is robust to outliers, thus we can tolerate erroneous points originated from previous steps. The RANSAC algorithm is initialized with two parameters: the maximum number of iterations and the minimum tolerance value used to assign a point to the inliers group. The maximum number of iterations can be obtained using the theoretical result from the original RANSAC paper [12].

(2) $$k=\frac{log(1-p)}{log(1-w^{n})}$$

where *p* is the probability of selecting a valid model in the presence of outliers and *w* is the probability of inliers in the dataset. In our study, we selected the number of iterations to be 100 and tolerance value to 1 and obtained less than 10 pixels error for our dataset even in the presence of noise, image distortion, and frame corruption.

**Figure 3:**
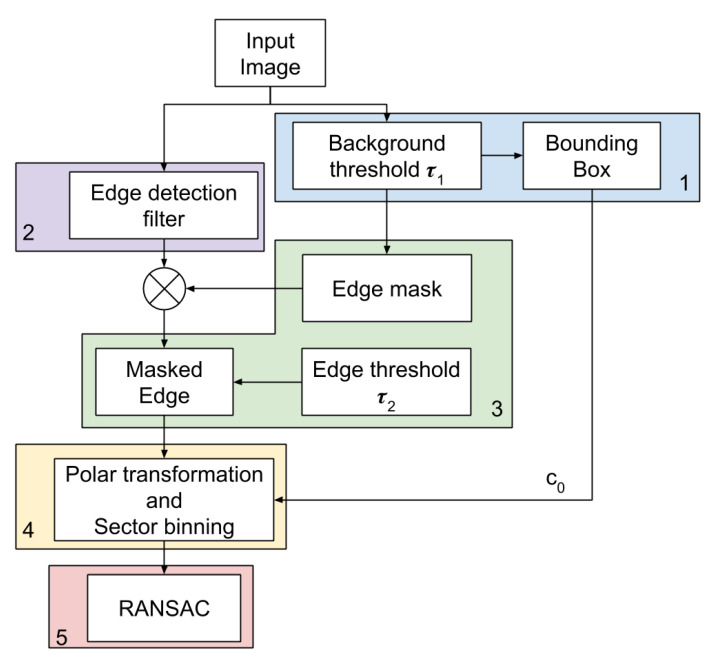
Procedure flowchart. The input image is processed by an edge detector filter and a simple background threshold with threshold parameter $\tau_{1}$. The binary output image obtained from the background segmentation is used to obtain the enclosing bounding box and estimate rough target centroid $c_{0}$. Also, the thresholded image is processed to obtain a binary edge mask using Equation (1). The edge image is thus masked and thresholded with tau2 in order to obtain limb point coordinates. Such coordinates are polar transformed relative to estimated centroid $c_{0}$. The sickle effect is handled by preserving only the coordinates with higher ρ value, whereas sector binning is used to remove images with points clustered in small arcs. Finally, Random Sample Consensus (RANSAC) algorithm performs radius and centroid fitting.

Furthermore, to improve the radius estimation we can perform Least Squares on the set of inliers returned by RANSAC. According to the results we obtained in our experiment, we believe that performing RANSAC is sufficient to achieve estimations with error < 10 pixels. The proposed algorithm is described in the block diagram in Figure 3.

**Figure 4:**
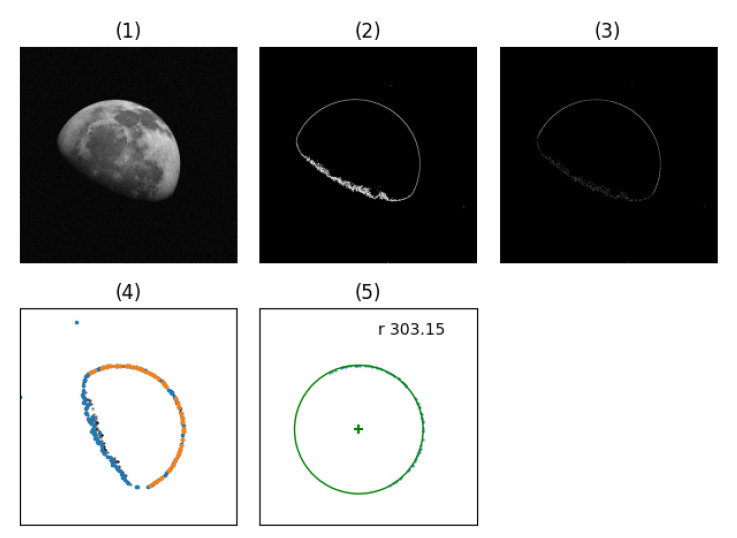
Complete image processing pipeline. (**1**) Input image corrupted with additive Gaussian noise; (**2**) binary edge mask resulting from Equation (3); (**3**) binary edge mask applied to result of edge detection, compared to (2) the resulting image contains the magnitude of the image gradient as floating point values. (**4**) Point cloud step. Points without sector binning are in black, dataset with sector binning is in blue and the set of inliers returned by RANSAC is in orange. (**5**) Final radius estimation with inliers.

In Figure 4 we show the output produced by each step of the pipeline.

## 4. Results

**Figure 5:**
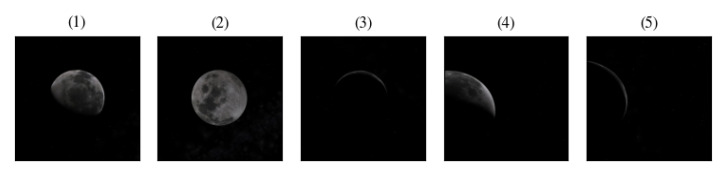
Examples images in the dataset. (**1**) Moon circle with terminator, (**2**) full disk, (**3**) scarce illumination, (**4**) partial frame occlusion, (**5**) partial frame occlusion with dim light.

To validate our method, we performed a set of experiments on a dataset of 50 synthetic 1024 × 1024 16-bit grayscale images of celestial bodies in different lighting conditions and positions, including partial frame occlusions (see Figure 5). The dataset has been generated with opensource software Celestia v1.6.1. We generated images of celestial bodies taken at various random distances, with the radius of the celestial body ranging from a minimum of 5 pixels to a maximum of 1024 pixels.

**Figure 6:**
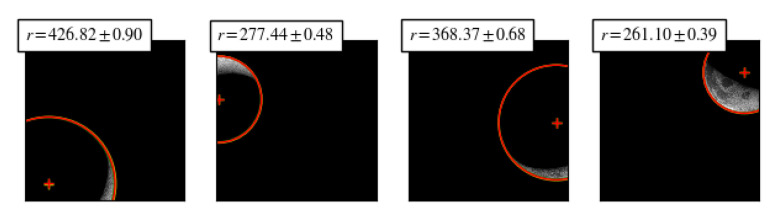
Radius estimation ± standard deviation for samples with the highest estimation variance.

We evaluated the accuracy of our method on the original dataset by performing a repeated radius estimation for each image. We reported the mean value and standard deviation for the target center and radius. For brevity, we present only a subset of relevant samples in Table 1. In Figure 6 we display qualitative results for some image samples.

We compared the radius and center estimation obtained by our method and Least Squares Estimation for circle identification. The effect of scarce illumination is particularly relevant for the Least Squares Estimator (LSE), whereas our RANSAC-based approach is able to provide better results for the same lighting conditions. For each image, we repeated the estimation 1000 times and reported mean value and standard deviation for the radius estimate and center coordinates. We performed the same experiment using LSE. It can be seen that LSE has a lower standard deviation than RANSAC for noise-free images; however, when we introduce noise LSE performance rapidly degrades. We performed a qualitative analysis to estimate the sensitivity of different sources of noise in the input image. We considered additive Gaussian noise with an increasing level of intensity, to simulate channel disturbances.

**Figure 7:**
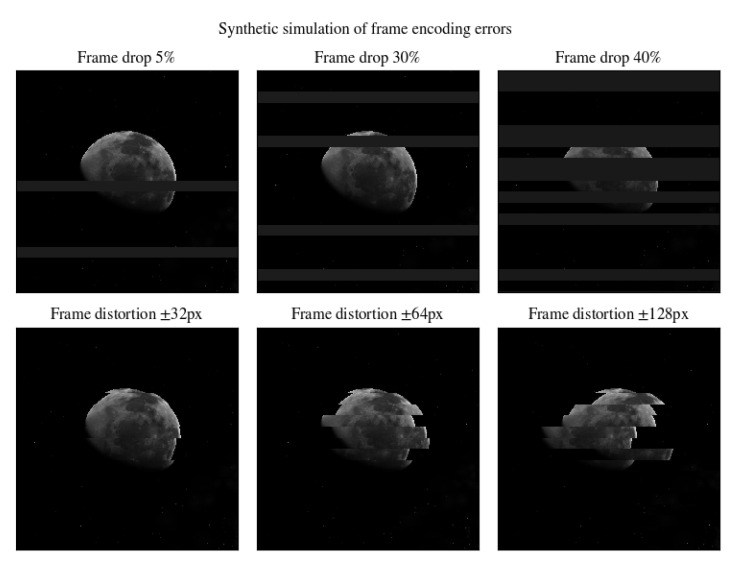
Images altered by various sources of noise.

**Figure 8:**
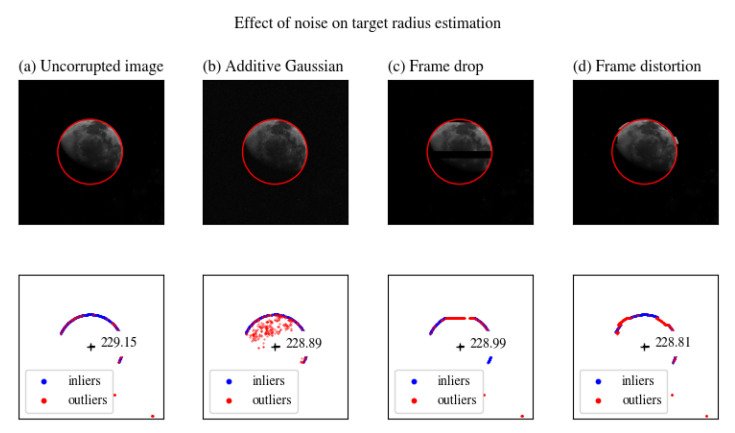
Output estimation for images corrupted by various sources of noise.

We also reproduced frame errors and data packet drop by altering and removing random image scanlines, see Figure 7 and Figure 8.

**Figure 9:**
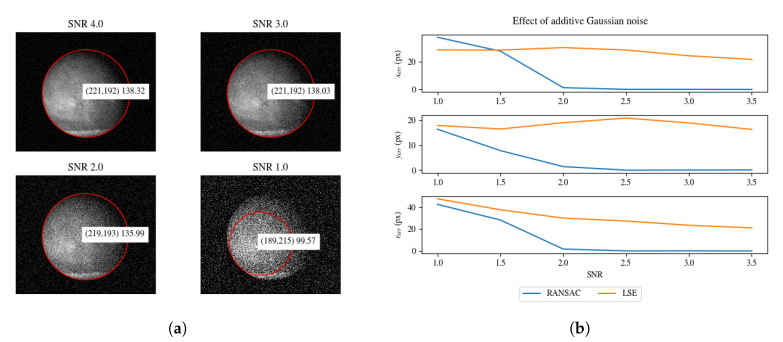
(**a**) Effect on parameter estimation for input image corrupted with additive Gaussian noise. (**b**) Model parameter estimation errors for increasing SNR. The error values *x_err_*, *y_err_* and *r_err_* are computed as the absolute difference between the parameter obtained from an uncorrupted image and the parameter obtained from the corrupted image.

We altered the original image with an additive Gaussian noise as shown in Equation (3), where $N(0,\sigma^{2})$ is a zero-mean Gaussian distribution with $\sigma^{2}$ variance (see Figure 9). We set the $\sigma^{2}$ variance to be equal to $SNR^{-1}$.

(3) $$I_{ij}=J_{ij}+N\left(0,\sigma^{2}\right)$$

In Figure 9, we reported the effect of additive Gaussian noise on the model parameter for increasing value of SNR. We compared the LSE model fitting to RANSAC. For SNR>2 (i.e., Gaussian noise with variance 0.5), RANSAC outperforms LSE significantly.

**Figure 10:**
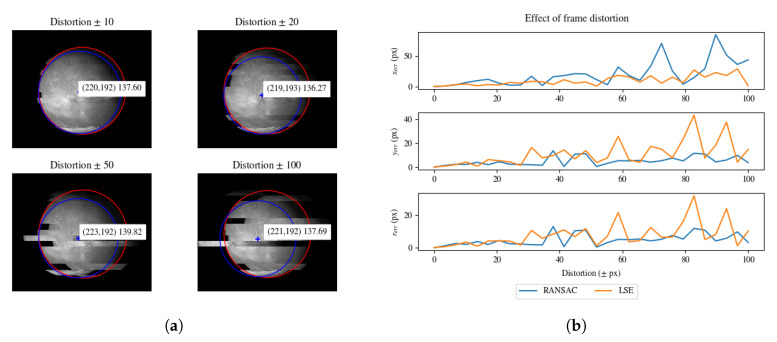
(**a**) Effect on parameter estimation for input image altered by local frame distortion. RANSAC estimation is in red, Least Squares Estimator (LSE) estimation is in blue. (**b**) Estimation errors for model parameters with frame distortion of an increasing amount of pixels.

We performed a similar analysis introducing local frame distortions (see Figure 10). The distortion value is the local translation of a random scanline by a certain amount of pixels.

We reported the effect of frame distortion errors in Figure 10. In general, RANSAC provides better results than LSE. However, it can be noted that the estimation error of the *x* coordinate is higher for RANSAC. The higher error in the x coordinate is related to the horizontal scanline translation.

**Figure 11:**
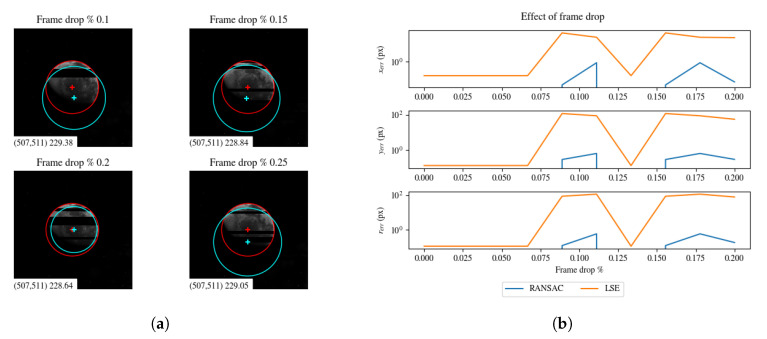
(**a**) Effect of frame drop in sample images. RANSAC estimation is in red, LSE estimation is in cyan. (**b**) Estimation error for an increasing percentage of frame drop. It can be noted the robustness of RANSAC with respect to LSE.

We simulated transmission errors by randomly dropping scanlines (see Figure 11). In Figure 11, we evaluated the effect of random scanline drop by comparing the estimation errors for RANSAC and LSE.

**Figure 12:**
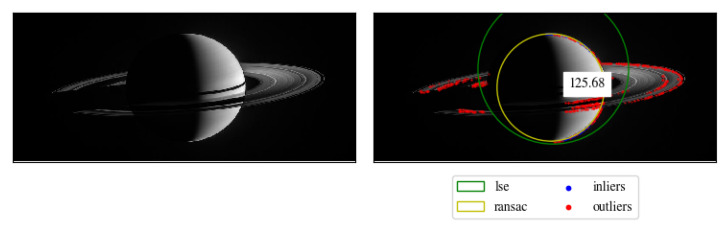
Estimation of Saturn radius comparison between RANSAC and LSE. RANSAC is able to provide accurate results even with a significant amount of outliers given by the rings of Saturn, whereas LSE is unable to provide satisfactory results.

Our RANSAC-based method can support various levels of partial occlusion, due to other celestial bodies or projected shadows. In Figure 12, we provided an example of the robustness of our approach compared to LSE. We observed that the rings of Saturn give rise to numerous outliers, moreover the projected shadows behave similarly to scanline drops.

Our pipeline depends on a small number of tunable parameters: background threshold $\tau_{1}$, edge threshold $\tau_{2}$, number of active sectors *S*, and total number of sectors *K* for the sector binning step, RANSAC number of iterations, and RANSAC tolerance value. The background threshold value $\tau_{1}$ can be estimated from the relative brightness of the target relative to the background. Generally, in space applications the target appears brighter than the background, thus the $\tau_{1}$ parameter can be easily obtained from the analysis of the image histogram. Small objects in the background that result after applying background thresholding are removed in the successive steps of the pipeline. Similarly, for edge threshold $\tau_{2}$, the threshold value can be obtained from the analysis of the magnitude of the image gradient. The edge threshold value is more relevant for the final estimation as it allows the detection of the target limb, therefore we performed grid-search with leave-one-out cross-validation to select the best value of $\tau_{2}$ for our dataset. The *K* parameter for sector binning allows the user to describe the circle as a composition of *K* sectors, thus *K* is related to the resolution of the sector binning. Since the previous steps of the pipeline produce a point cloud that spans over a small arc of the total circle, the *S* parameter is used to represent the confidence level for the set of points to be included in the final RANSAC estimation. Both parameters *K* and *S* have been selected performing grid-search leave-one-out cross-validation. Finally, the RANSAC parameter *number of iterations* can be computed analytically from Equation (2), whereas the RANSAC tolerance value has been selected to a very low value in order to obtain accurate estimation.

We expect our pipeline to be implemented on an onboard computer for spacecraft navigation, possible with constraints on maximum execution time to process an image. Thus, we report in Table 2 the relative amount of processing time required for each step of the pipeline. The most intensive step in the pipeline is the RANSAC step that is responsible for almost 45% of the total execution time.

The total execution time, based on a set of 500 trials, on a softcore LEON3 (grlib-gpl 2019.2 b4241) implemented on Virtex6 Field Programmable Gate Array (FPGA) (Development board ML605 REV E) is 542.22 ± 4.90 ms. The RANSAC step execution time only is approximately 244 ms. As reported in [23], CPU-only image processing may incur unacceptable latency, thus we advocate the implementation of time-critical steps in the pipeline on FPGA or Commercial off-the-shelf (COTS) systems. We should also note that we considered a single core processing unit. If the system provides support for parallel computing or vectorized instructions, the execution time of more than one step in the pipeline can be improved. In particular, the first image processing operations (steps 1–3) can be fully parallelizable. Furthermore, some operations in steps 4 and 5 can also be run in parallel in order to accelerate the frame processing time.

## 5. Conclusions

In this paper, we presented a robust image processing pipeline for estimating the center and radius of planets and moons that can be integrated into an Autonomous Navigation System for modern spacecraft. We evaluated the resilience of our method to various sources of noise and reported appreciable results handling images with significant amount of noise, frame distortions, artifacts, and partial occlusions. We reported the pixel-level accuracy and the effect of noise on the estimated target parameters. We aimed at a computationally low-demanding pipeline able to be run on an onboard computer with limited resources.

The presented method allows performing radius and center estimation of planets on board space-qualified processors, such as LEON3, with acceptable latency. This fact is fundamental to perform Visual-Based Navigation for missions having strict requirements in terms of radiation tolerance, such as Medium-Earth Orbit, Geostationary Earth Orbit, or long-term Low Earth Orbit missions [24,25,26].

However, despite the high immunity to outliers and noise, the applicability of the methods is limited to planets, whose projection on the image plane is a circle. A complex task for future research involves the pose estimation of non-collaborative objects having an arbitrary shape. The complexity of this task is enhanced by the lack of information on the attitude and positions, and on the noise, outliers, occlusions, motions, and others. In addition, the complexity of the algorithms used in these applications is often prohibitive given the strict power consumption and memory footprint requirements of space-qualified platforms [24]. For instance, one of the most promising approaches involves the use of Deep Neural Networks [27], which demonstrated promising potentials for this kind of application. However, the high computational cost required to perform the inference of Deep Neural Networks often forces the use of modern Commercial off-the-shelf hardware accelerators featuring higher computational efficiency than space-qualified devices. Although Deep Neural Networks proved to perform complex tasks and can be easily accelerated with COTS hardware accelerators, their applicability in long-term missions is still under extensive investigation, and it currently poses an important open research question [24,28,29].

## 6. Future Works

In future works, we expected to extend our pipeline to support for multi-target detection and parameter estimation for non-ellipsoidal objects such as comets and asteroids. Furthermore, we planned to implement FPGA-based hardware accelerators for the different blocks of the proposed pipeline to further reduce the processing latency.

## Figures and Tables

**Table 1:** Estimation mean error and standard deviation for target center (x,y) coordinates and radius for some samples in the dataset.

Filename	${\mathrm{err}}_{x}$	${\mathrm{err}}_{y}$	${\mathrm{err}}_{r}$
0001	0.074 ± 0.050	0.084 ± 0.064	0.067 ± 0.040
0002	0.719 ± 0.566	0.147 ± 0.110	0.715 ± 0.550
0014	0.268 ± 0.158	0.137 ± 0.087	0.207 ± 0.124
0027	0.103 ± 0.058	0.176 ± 0.164	0.155 ± 0.126
0030	0.159 ± 0.106	0.040 ± 0.046	0.127 ± 0.094
0041	0.107 ± 0.086	0.051 ± 0.043	0.074 ± 0.044
0045	0.115 ± 0.050	0.054 ± 0.047	0.067 ± 0.058
0049	0.326 ± 0.149	0.362 ± 0.103	0.361 ± 0.139
0050	0.105 ± 0.063	0.126 ± 0.092	0.134 ± 0.107

**Table 2:** Relative amount of execution time for each step in the processing pipeline.

Pipeline Step	%
Background threshold	0.57
Bounding box	17.32
Morphological Edge mask	15.48
Edge detection	17.71
Masked edge	1.67
Polar transf. and Sector binning	1.33
RANSAC	45.92
